# Enterococcus faecium Empyema Following Extracorporeal Membrane Oxygenation for COVID-19 Acute Respiratory Distress Syndrome

**DOI:** 10.7759/cureus.42789

**Published:** 2023-08-01

**Authors:** Mark R Brown, Joshua M Boster, Stephen M Goertzen, Michael J Morris, Erik S Manninen

**Affiliations:** 1 Internal Medicine, San Antonio Military Medical Center, San Antonio, USA; 2 Pulmonary and Critical Care Medicine, San Antonio Military Medical Center, San Antonio, USA; 3 Pulmonary and Critical Care, San Antonio Military Medical Center, San Antonio, USA; 4 Critical Care Medicine, San Antonio Military Medical Center, San Antonio, USA

**Keywords:** vv ecmo, veno-venous ecmo, anticoagulation, enterococcus faecium, pleural empyema, covid-19, ecmo

## Abstract

A 33-year-old male with severe COVID-19 required prolonged veno-venous extracorporeal membrane oxygenation (ECMO) support. Following decannulation, he developed an *Enterococcus faecium *empyema. Tube thoracostomy and broad-spectrum antibiotics were initiated, followed by an unsuccessful attempt at pleural irrigation with saline, given the patient had an increased risk of bleeding due to the concomitant requirement for systemic anticoagulation. Subsequently, intrapleural tissue plasminogen activator (tPA) and recombinant human Dornase alfa (DNase) were safely administered with the resolution of empyema. *Enterococcus faecium *is an uncommon cause of pleural empyema and, to our knowledge, has not previously been reported to be associated with COVID-19 or ECMO.

## Introduction

The complications of coronavirus disease 2019 (COVID-19) infections have been well-described over the past several years, including pleural effusion, pneumothorax, and rarely empyema, which is associated with a significantly increased rate of morbidity and mortality [1]. Empyemas are typically associated with organisms such as *Streptococcus pneumoniae*, *Staphylococcus aureus*, *Escherichia coli*, and *Bacteroides fragilis*; rarely, bacteria such as *Enterococcus faecium* have been documented in such cases [2-5].

The usual management of pleural empyema requires both antibiotic therapy and drainage [6]. When drainage cannot be readily achieved with tube thoracostomy, the use of adjuncts such as pleural saline irrigation or the addition of tissue plasminogen activator (tPA) and recombinant human Dornase alfa (DNase) may be required [7,8]. The safety of the latter treatment modality has not been well documented in patients who are systemically anticoagulated. Here we present a case of *Enterococcus faecium *empyema in a patient with COVID-19 after extracorporeal membrane oxygenation (ECMO). He ultimately required intrapleural tPA/DNase while systemically anticoagulated after saline irrigation, which was unsuccessful.

## Case presentation

A 33-year-old Hispanic male with morbid obesity presented with acute respiratory distress syndrome (ARDS) secondary to COVID-19. He was intubated 10 days after symptom onset and subsequently placed on veno-venous ECMO after failing to improve despite maximum ventilator support, prone positioning, neuromuscular blockade, and inhaled pulmonary vasodilators. His treatment included remdesivir, dexamethasone, and tocilizumab. His course was complicated by right heart failure requiring mechanical support, acute renal failure requiring hemodialysis, and a superior vena cava thrombus. During his hospitalization, he was successfully treated for multiple nosocomial infections, including bacteremia with methicillin-sensitive* Staphylococcus aureus*, *Klebsiella pneumoniae*, and ampicillin-sensitive *Enterococcus faecalis*, as well as ventilator-acquired pneumonia (VAP) secondary to *Pseudomonas aeruginosa *and *Klebsiella pneumoniae*. He was decannulated after 130 days while still requiring mechanical ventilator support via tracheostomy.

**Figure 1:**
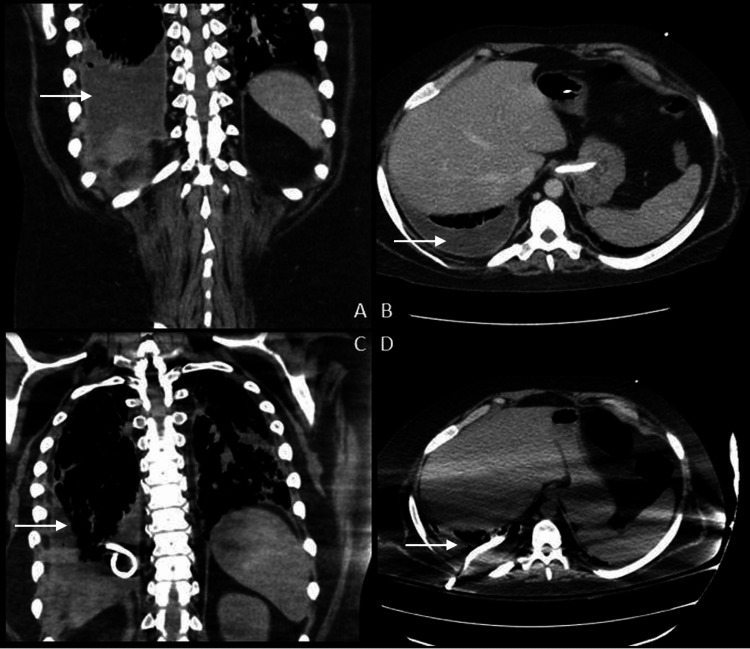
A computed tomography scan of the pleural empyema before and after drainage Figures 1A and 1B were taken prior to the insertion of the pigtail catheter. Figures 1C and 1D were taken after six days of treatment with tPA/DNase. Arrows point to the location of the empyema.

Sixteen days after decannulation, the patient developed respiratory distress as well as signs concerning an emerging infection. Vancomycin and meropenem were initiated, and a CT scan of the chest, abdomen, and pelvis was obtained that demonstrated a right pleural effusion with concern for empyema (Figures 1A, 1B).

Pleural sampling was grossly purulent and consistent with empyema (Table 1).

**Table 1:** Pleural effusion cytology results

Pleural Fluid Analysis	
Color	Red
Appearance	Cloudy
Cell Count	154,900
Red Blood Cells/mcL	287,000
Nucleated Cells/mcL	155,260
Segmented Neutrophils (%)	78.2
Mononuclear Cells (%)	21.8
Glucose (mg/dL)	<5
Protein (g/dL)	4.9
Lactate Dehydrogenase (U/L)	>200

A 14-French chest tube was placed, and pleural irrigation with normal saline was initially attempted without success. Subsequently, in the setting of systemic anticoagulation, intrapleural TPA/Dornase was initiated with close monitoring; the patient demonstrated radiographic improvement after a total of six days of therapy (Figures 1C, 1D). Pleural fluid cultures and sensitivities revealed an ampicillin-sensitive *Enterococcus faecium*, and he was continued on a six-week course of ampicillin with the resolution of the empyema.

## Discussion

Extracorporeal membrane oxygenation (ECMO) has been extensively used during the COVID-19 pandemic. The CESAR (conventional ventilatory support vs. extracorporeal membrane oxygenation for severe adult respiratory failure) and EOLIA (ECMO to rescue lung injury in severe ARDS) trials showed that early evaluation and initiation of ECMO were associated with significant improvement in patient outcomes compared to conventional management [9,10]. Bleeding and thrombotic complications are the leading causes of morbidity and mortality associated with ECMO, and in patients who are anticoagulated, the utilization of thrombolytic medications is typically avoided [11,12]. Intrapleural empyema has not been directly associated with ECMO patients, and to our knowledge, no cases have been reported in which a patient who required ECMO support after COVID-19 subsequently developed an *Enterococcus faecium* intrapleural empyema.

*Enterococcus faecium* is a gram-positive, facultative anaerobic cocci that is a commensal gastrointestinal organism and rarely associated with intrapleural infections [13]. A study of 110 serious infections due to the *Enterococcus *genus over the course of one year across six hospitals found only 4% of those infections were located in the respiratory tract [14]. *Staphylococcus aureus* and *Pseudomonas aeruginosa* are among the most common culture-identified organisms in patients with hospital-acquired empyema [15]. Apart from morbid obesity, our patient did not have any additional comorbidities such as alcoholism, diabetes, or gastroesophageal reflux disease that would put him at higher risk for gastrointestinal microbiota-associated empyema [16]. Prior reports of *Enterococcus faecium* empyema have primarily been associated with fistulization between the gastrointestinal system and the pleural space; however, our patient had none [3,5]. Our patient did have* Enterococcus faecaelis *bacteremia earlier in his hospitalization while on ECMO, which resolved with the administration of ampicillin; however, this empyema revealed the first *Enterococcus faecium*-positive culture.

Standard treatment of empyema consists of broad-spectrum empiric antibiotics to cover common organisms and pleural drainage via tube or catheter; in approximately one-third of patients, surgical intervention may be required [17]. The pleural irrigation trial (PIT) showed that the addition of saline irrigation in addition to standard management resulted in a radiographically significant reduction in pleural fluid volume [8]. This approach is both affordable and safe for patients who are systemically anticoagulated. The MIST2 (second multicentre intrapleural sepsis trial) trial demonstrated that the utilization of tPA and DNase was associated with a decreased length of hospital stay and a reduction in the need for surgical intervention; however, the majority of the patient population in that study was not systemically anticoagulated [7]. To the best of our knowledge, no large randomized controlled trials have been conducted that compare intrapleural saline irrigation (PIT) to tPA/DNase administration (MIST2). Our patient was originally trialed with the PIT protocol but failed to improve. After the administration of tPA/DNase, there was radiographic evidence of the resolution of the empyema. In patients with a high risk for bleeding, such as anticoagulation, thrombolytics are typically avoided as there is little data available regarding safety [18,19]. Our patient did not experience any significant adverse events, suggesting that intrapleural fibrinolytics may be a reasonable option, when necessary, for patients who require systemic anticoagulation.

## Conclusions

*Enterococcus faecium *is an unusual cause of empyema that has not been previously reported in association with ECMO or COVID-19. The use of intrapleural tPA/DNase for patients with complicated empyema that fails to improve with tube thoracostomy and pleural irrigation is reasonable even in the setting of a concomitant requirement for systemic anticoagulation. 
